# A standard curve based method for relative real time PCR data processing

**DOI:** 10.1186/1471-2105-6-62

**Published:** 2005-03-21

**Authors:** Alexey Larionov, Andreas Krause, William Miller

**Affiliations:** 1 Breast Unit, Western general Hospital, Edinburgh, UK; 2 Novartis Pharmaceuticals, Biostatistics, CH – 4002 Basel, Switzerland; 3 Breast Unit, Edinburgh University, Edinburgh, UK

## Abstract

**Background:**

Currently real time PCR is the most precise method by which to measure gene expression. The method generates a large amount of raw numerical data and processing may notably influence final results. The data processing is based either on standard curves or on PCR efficiency assessment. At the moment, the PCR efficiency approach is preferred in relative PCR whilst the standard curve is often used for absolute PCR. However, there are no barriers to employ standard curves for relative PCR. This article provides an implementation of the standard curve method and discusses its advantages and limitations in relative real time PCR.

**Results:**

We designed a procedure for data processing in relative real time PCR. The procedure completely avoids PCR efficiency assessment, minimizes operator involvement and provides a statistical assessment of intra-assay variation.

The procedure includes the following steps. (I) Noise is filtered from raw fluorescence readings by smoothing, baseline subtraction and amplitude normalization. (II) The optimal threshold is selected automatically from regression parameters of the standard curve. (III) Crossing points (CPs) are derived directly from coordinates of points where the threshold line crosses fluorescence plots obtained after the noise filtering. (IV) The means and their variances are calculated for CPs in PCR replicas. (V) The final results are derived from the CPs' means. The CPs' variances are traced to results by the law of error propagation.

A detailed description and analysis of this data processing is provided. The limitations associated with the use of parametric statistical methods and amplitude normalization are specifically analyzed and found fit to the routine laboratory practice. Different options are discussed for aggregation of data obtained from multiple reference genes.

**Conclusion:**

A standard curve based procedure for PCR data processing has been compiled and validated. It illustrates that standard curve design remains a reliable and simple alternative to the PCR-efficiency based calculations in relative real time PCR.

## Background

Data processing can seriously affect interpretation of real time PCR results. In the absence of commonly accepted reference procedures the choice of data processing is currently at the researcher's discretion. Many different options for data processing are available in software supplied with different cyclers and in different publications [1-7]. However, the basic choice in relative real time PCR calculations is between standard curve and PCR-efficiency based methods. Compared to the growing number of studies addressing PCR efficiency calculations [3,5,8-10] there is a shortage of publications discussing practical details of the standard curve method [11]. As a result, the PCR efficiency approach appears as the method of choice in data processing for relative PCR [12]. However, when reliability of results prevails over costs and labor load, the standard curve approach may have advantages.

The standard curve method simplifies calculations and avoids practical and theoretical problems currently associated with PCR efficiency assessment. Widely used in many laboratory techniques this approach is simple and reliable. Moreover, at the price of a standard curve on each PCR plate it also provides the routine validation for methodology. To benefit from the advantages of the standard curve approach and to evaluate its practical limitations we designed a data processing procedure implementing this approach and validated it for relative real time PCR.

## Results

### Description of the data processing procedure

#### Source data

Raw fluorescence readings were exported from Opticon Monitor software and processed in MS Excel using a VBA script (the mathematical formulae, script and samples of source data are attached to the electronic version of publication, see Additional files 1 and 2).

#### Noise filtering

**Figure 1:**
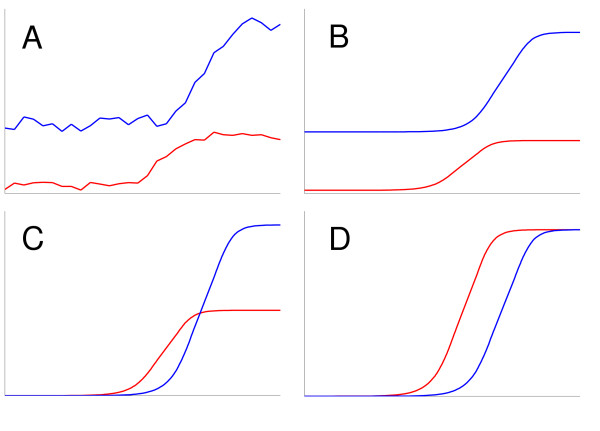
**Noise filtering**. Axes: vertical – fluorescence, horizontal – cycle number, A Source data, B Smoothing, C Baseline subtraction, D Amplitude normalization

The random cycle-to-cycle noise was reduced by smoothing with a 3 point moving average (two-point average in the first and the last data points). Background subtraction was performed using minimal value through the run. If significant scattering in plateau positions was observed it was removed by amplitude normalization (normalizing by maximal value in the cell over the whole PCR run). The noise filtering is illustrated in the Figure 1.

#### Crossing points calculation

**Figure 2:**
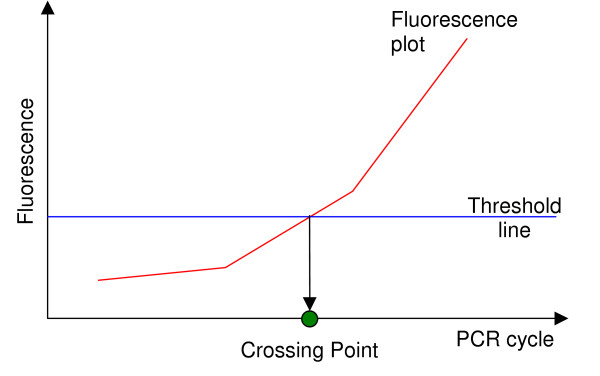
Direct calculation of crossing points.

The crossing points (CPs) were calculated directly as the coordinates of points in which the threshold line actually crossed the broken lines representing fluorescence plots obtained after the noise filtering (Figure 2). If several intersections were observed the last one was used as the crossing point.

#### Standard curve calculation

A standard curve was derived from the serial dilutions by a customary way. Relative concentrations were expressed in arbitrary units. Logarithms (base 10) of concentrations were plotted against crossing points. Least square fit was used as the standard curve.

#### Threshold selection

The optimal threshold was chosen automatically. The VBA script examined different threshold positions calculating coefficient of determination (r^2^) for each resulting standard curve. The maximum coefficient of determination pointed to the optimal threshold (typically the maximum r^2 ^was larger than 99%).

#### Calculating means and variances of means for crossing points in PCR replicas

The optimal threshold was used to calculate CPs for unknown samples. Means and variances of means were then calculated for CPs in PCR replicas.

#### Derivation of non-normalized values from crossing points

The non-normalized values were calculated from the CPs' means by the standard curve equation followed by exponent (base 10). The variances were traced by the law of error propagation.

#### Summarizing data from several reference genes to a single normalizing factor

Two options are available in the VBA script to summarize data from multiple reference genes:

- Arithmetic mean (deprecated),

- Geometric mean (recommended).

#### Calculation of normalized results for target genes

The final results representing relative expression of target genes were calculated by dividing the non-normalized values by the above normalization factor. The normalized results' variances were derived by the law of error propagation.

When confidence intervals or coefficients of variation were needed they have been calculated from the corresponding variances (see Additional file 1 with formulae for details).

### Procedure testing and validation

**Figure 3:**
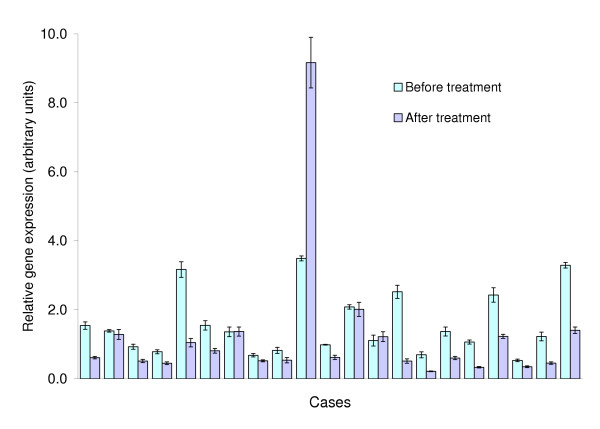
**Expression of Cyclin B1 mRNA in breast cancer biopsies**. The observed decrease of Cyclin B1 expression after treatment was expected in most but not all cases. Bars show actual 95% confidence intervals estimated by the described statistical procedure in a set of real clinical specimens (*NB *– these are confidence intervals for intra-assay PCR variation only).

We tested this procedure on the measurement of expression of 6 genes in 42 breast cancer biopsies (Figure 3, Table 1).

**Figure 4:**
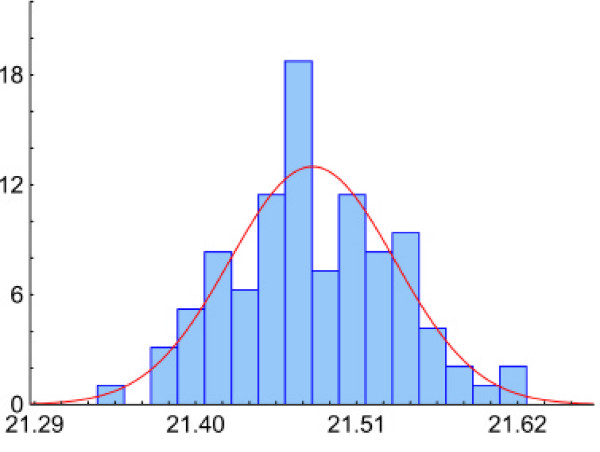
**Distribution of crossing points in PCR replicas**. Axes: vertical – relative frequency (%), horizontal – crossing points. Histogram represents a typical crossing points' distribution in 96× replica (Plate 1 from Table 2). The Kolmogorov-Smirnov test has not revealed significant deviations from the Normal distribution. The red line shows a Normal fit.

To validate the assumption of a Normal distribution for the initial data (*i.e*. CPs) we studied distributions of crossing points in four plates, each of which represented a 96× PCR replica. The observed distributions were symmetric, bell-shaped and close to a Normal distribution (Figure 4, Table 2).

**Figure 5:**
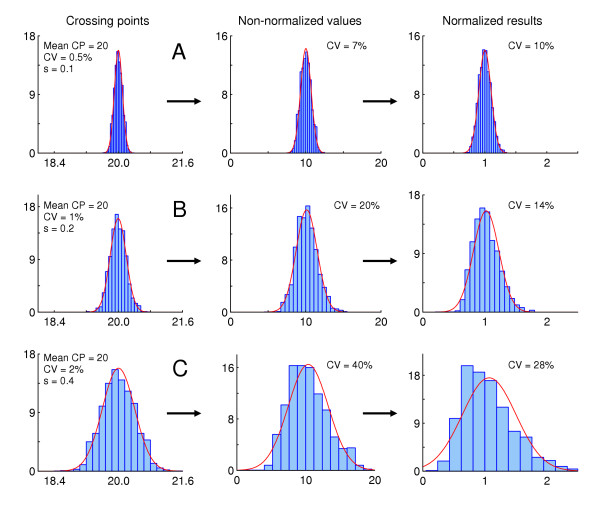
**Transformation of normal distribution through data processing**. Axes: vertical – relative frequency (%), horizontal – results. Red lines show Normal fits. **A: **At CPs' CV 0.5% the deviations from normality were not detectable using the Kolmogorov-Smirnov test. **B: **At CPs' CV 1% the deviations from normality were not detectable in non-normalized values though moderate deviations were detectable in final results. **C: **At CPs' CV 2% deviations from normality were detectable in both non-normalized values and in final results.

Transformation of the Normal distribution through PCR data processing was analyzed by a computer simulation. It showed that the shape of resulting distributions significantly depends on the initial data dispersion. At low variation in crossing points (SD < 0.2 or CV < 1%) the distributions remain close to Normal through all steps of data processing (Figure 5-A). In contrast, at higher initial dispersion (crossing points' SD > 0.2 or CV > 1%) the PCR data processing transformed the Normal distribution such that the resulting distributions became asymmetric and far from normal (Figure 5-C).

**Figure 6:**
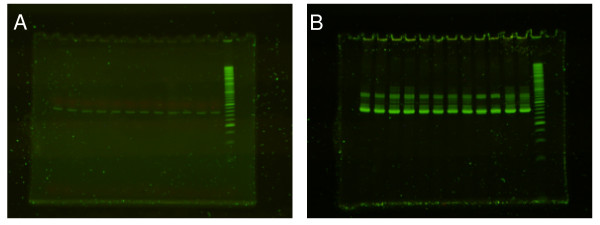
**Effect of staining with SYBR Green 1 on PCR gel**. A: Before staining. B: After staining. Before electrophoresis SYBR Green1 was added to marker but not to samples.

**Figure 7:**
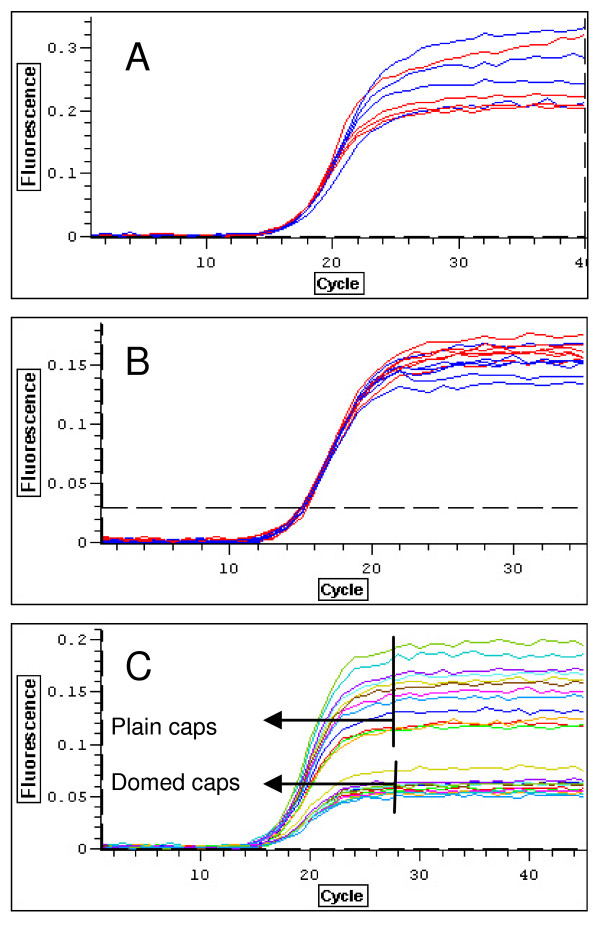
**Effect of different factors on plateau position**. A: More enzyme in blue than in red samples B: More primers in blue than in red samples C: Domed and plain caps

Addressing the use of amplitude normalization we studied several factors potentially affecting PCR plateau level. On the gels run immediately after PCR the weak bands initially visible without staining because of SYBR Green originated from PCR mixes were remarkably increased after additional staining with SYBR Green (Figure 6). When PCRs were run with different concentrations of primers, enzyme, and using different caps for PCR plate, neither increase of primers nor addition of enzyme influenced the plateau level and scattering. However, the caps design did affect the plateau position (Figure 7).

## Discussion

PCR data processing is a complex procedure that includes a number of steps complementing each other. Many different options have been suggested by different authors at each step of PCR data processing. In the discussion below we go through our procedure on a step-to-step basis shortly discussing the available options and explaining our choices. In general, we preferred the simplest functioning solutions. In statistical treatment we looked for valid practical estimations rather than for mathematically exact solutions. Because of lack of relevant theoretical data we paid especial attention to the amplitude normalisation and to statistical processing of intra-assay PCR replicas. To validate these sections of our procedure we had to address some basic theoretical issues.

PCR data processing may need to be optimized for specific PCR machines and chemistry. The discussed processing was optimized for data obtained on an Opticon Monitor 2 machine (MJ Research) using the QuantiTect SYBR Green PCR kit (Qiagen).

### Smoothing

Smoothing is necessary if noticeable non-specific scattering from cycle to cycle is observed on the raw fluorescence plots. Apart from moving averages there are other more sophisticated mathematical approaches to filter this kind of noise *e.g*. sigmoidal fitting [13]. However, this fit is no more than a mathematical abstraction fitting PCR plot. Until the development of a genuine mathematical model of real time PCR, all other fits will not be related to PCR *per se*. Therefore, since simple 3 point moving average produced acceptable results there was no obvious need for more complex methods.

### Background subtraction

Background subtraction is a common step in PCR data processing. Often it requires operator's involvement to choose between several available options (*e.g*. subtraction of minimal value through the run, subtraction of average over a certain cycle ranges, different kinds of "trends", *etc*). To avoid the operator involvement we always subtract the minimal value observed in the run. This option has a clear interpretation and works well. It is important that the baseline subtraction is performed *after *smoothing. So the noise potentially affecting minimal values has already been reduced before baseline subtraction.

### Amplitude normalization

Amplitude normalization unifies plateau positions in different samples. Although amplitude normalization was available in some versions of Light-Cycler software and has been used by some researchers [14] this step still is not common in PCR data processing. The caution with regard to the amplitude normalization is probably caused by current lack of understanding of the plateau phase in PCR.

Amplitude normalization is based on the suggestion that in ideal PCR, output is determined by the initially available PCR resources. In this case PCRs prepared from the same master mix will run out of the same limiting resource in different samples. The resource can run out sooner (abundant template) or later (rare template) but finally the same amount of PCR products will be produced in all samples. This assumption is valid for ideal PCR but in practice it may not always hold (for example, non-specific PCR products may also consume PCR resources). The factors potentially leading PCR to the plateau include utilization of primers or nucleotides, thermal inactivation of DNA polymerase, competition between primers and PCR products for annealing, enzyme inactivation by PCR products and accumulation of inhibitors [15]. The plateau may also be affected by factors influencing the detection of PCR products: *e.g*. by PCR volume and by concentration of probe or SYBR-Green in PCR mix [14,16,17]. In practice the plateau phase is probably caused by different factors depending on the particular PCR design and PCR mix composition.

In this work we used QuantiTect SYBR Green PCR kit (Qiagen). With this kit neither increase of primers nor addition of enzyme notably affected the plateau positions (Figure 7). The fact that bands on PCR gels were remarkably enlarged by additional staining with SYBR Green (Figure 6) suggests that the plateaus observed in PCRs could had been caused simply by limited SYBR Green concentration. Therefore, in samples prepared with the same master mix, the plateau scattering could be considered as a non-specific noise and should be removed.

**Figure 8:**
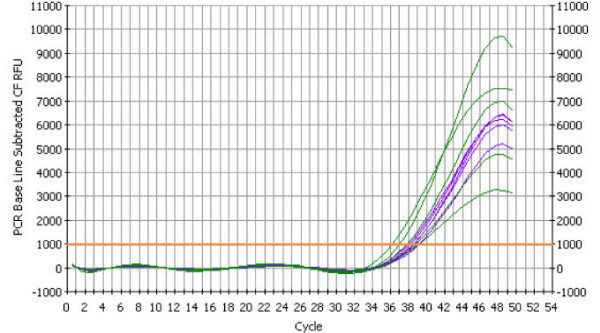
**Optical factors affect the plateau scattering**. SYBR Green real time PCR in frosted plates (green) and white plates (blue). Frosted plates cause increased plateau scattering because of inconsistent reflection and refraction (Reproduced from [18], with ABgene^® ^permission).

What may cause the plateau scattering in fluorescence plots? In certain cases, it may be optical factors. Freshwater *et al *[18] showed that refraction and reflection notably affects the plateau scattering in different types of tubes (Figure 8). This is in agreement with our observations in which (i) we failed to observe positive correlation between plateau positions and the volumes of bands on PCR gels and (ii) plateau scattering may be reduced by passive dye normalization (data not shown). Potentially, other factors may also play a role in plateau scattering: *e.g*. non-uniform evaporation across PCR plates[18].

**Figure 9:**
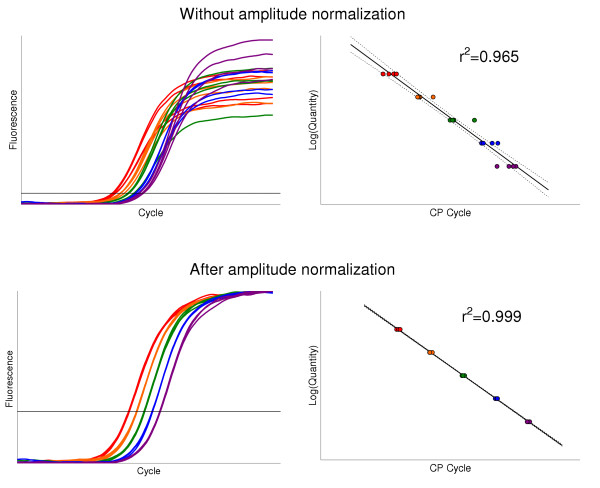
Effect of amplitude normalization on standard curve.

So far, lack of understanding of the PCR plateau nature makes the amplitude normalization an optional step. When used, amplitude normalization should be empirically validated in each individual plate. Linearity of the standard curve may act as an empirical test for amplitude normalization, *i.e*. if the standard curve is good so the amplitude normalization does not alter the results and the procedure may be employed. Our experience is that amplitude normalization usually improves the standard curve (Figure 9).

**Figure 10:**
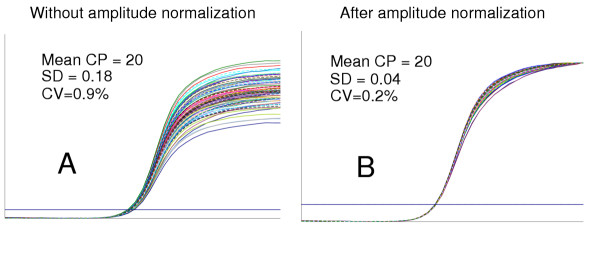
**Effect of amplitude normalization on plateau scattering in 96× replica**. Axes: vertical – Fluorescence, horizontal – Cycle. Data for plate 3 from Table 2.

Finally, a "PCR-specific" explanation of plateau scattering can not explain the scattering observed in PCR replicas (Figure 10A). After amplitude normalization the fluorescence plots in replicas often converge toward a single line (Figure 10B). In our experiments this reduced CV in replicas by a factor of 2 to 7. Therefore, when a marked plateau scattering is observed at a particular PCR, amplitude normalization should be considered.

### Threshold selection

As long as the standard curve provides both basis and empirical validation for PCR results the threshold may be put at any level where it produces a satisfactory standard curve. At the same time, the linearity of standard curve is theoretically explained at exponential phase of PCR only. Therefore, the common practice is to put the threshold as low as possible to cross the fluorescence plots in the exponential phase. For this reason we usually restrict the search of the optimal threshold position to the lower half of the fluorescence plot.

### Crossing point calculation

Currently the most established methods of crossing point calculations are the fit point method and the second derivative maximum method [4]. The fit point method reliably allocates the threshold level in the exponential phase and reduces minor inaccuracies by aggregating data from several points. The second derivative maximum method eliminates interactivity during threshold selection and baseline subtraction. These are robust and reliable methods.

Our calculation method also produces good results. In addition, it is simple and does not alter the initial mathematical definition of crossing points.

### Statistical treatment of PCR replicas

The next step in the data processing is derivation of results from crossing points. Two separate issues need to be addressed during this step: (i) best-fit values and (ii) errors in replicates. Calculation of best-fit values is simple with standard curve methodology (see formulae in Additional file 1) but statistical assessment of errors in replicates requires detailed consideration.

#### Description and interpretation of intra-assay PCR variation

PCR uncertainty is usually characterized by coefficient of variation. This reflects the fact that the errors propagated to non-normalized values and to final results are higher at higher best-fit values. This is not always the case with the crossing points. However, coefficients of variation still may be used for rough comparison of CPs' dispersions because the CPs' absolute values vary in quite a limited range (typically between 20 and 30 cycles).

Importantly, that during PCR interpretation the statistical significance of differences between samples should not be based on intra-assay variation. Intra-PCR replicates account only for errors originated from PCR. At the same time the uncertainty in final results is usually more affected by pre-PCR steps [1]. In this case the replicates of the whole experiment (including sampling, RNA extraction and reverse transcription) are needed to derive statistical differences between samples. If the amount of starting material is limited or replicates are unavailable (for example when studying tumor biopsies) the preliminary assessment of replicates in an experimental set of similar samples is required to base statistical comparison between samples (type B evaluation of uncertainty according to Taylor and Kuyatt [19]). This type of statistical treatment is not included in the described data processing. Even though in our experiments the intra-assay PCR variation can not be directly used for statistical inferences, we routinely use it as an internal quality check for PCR.

#### Starting point for statistical assessment

Two different approaches may be utilized for initial statistical handling of intra-assay PCR replicates. Either CP values are first averaged and then transformed to non-normalized values or *vice versa*. Both approaches may yield similar results, as long as the arithmetic mean is used for the CP values and geometric mean for the non-normalized quantities. We prefer to start statistical assessment using unmodified source data *i.e*. we average crossing points before transformation to the non-normalized values.

#### Crossing point distribution in PCR replicas

To choose appropriate statistical methods to deal with crossing points, we started from the assessment of crossing points' distributions in PCR replicates. Distributions of crossing points were studied in four PCR plates each of those represented a 96× replicate. The distributions were close to the Normal (Table 2, Figure 4). Combined analysis of a number of PCR reactions, made in triplicates or quadruplicates, confirmed this result (data not shown). Therefore, Normal distribution satisfactorily reflects the distribution of crossing points in PCR replicates. This allowed us to use arithmetic mean and mean's variances to estimate best-fit values and their uncertainty in crossing points.

#### Error propagation

The CPs' variances were traced to final results by the law of error propagation. This assumed the normality of distributions not only in crossing points but also at the later steps of data processing. Strictly speaking, this assumption is not completely true: the data processing deforms normal distribution. Three functions are used to calculate results from crossing points: linear function (linear standard curve), exponent (calculation of non-normalized values) and ratio (normalizing by reference genes). Among them only linear function keeps normality of distribution. Exponent and ratio distort it. At the same time, the degree of the introduced distortion depends on particular numeric parameters. Analyzing the deformation of normal distribution at the parameters typical for real time PCR we found that at low initial dispersions the resulting distributions remain close to normal (Figure 5A). Therefore, the convenient parametric methods can be used in PCR data processing if crossing points' CV in replicas does not exceed 1% (for a typical PCR it roughly corresponds to crossing points' SD ≤ 0.2 and to CV in non-normalized values ≤ 14%, see Table 3). At higher initial dispersions the resulting distributions become asymmetric and require special statistical treatment (Figure 5C). Actually observed in our experiments crossing points' CVs usually were less than 0.5% (Table 2).

Additionally the analysis confirmed the remarkable increase of relative variation at each step of data processing. *E.g*. 2% CV at crossing points resulted to 28% CV in the non-normalized values and to 40% CV in the final results (Table 3). This also complicates interpretation of results with high dispersion in crossing points.

#### Standard curves

In line with the common practice, we interpreted the standard curve as an ordinary linear function ignoring its statistical nature and uncertainty because the uncertainty was usually quite small (typical coefficient of determination above 99%). With sufficient number and range of standard dilutions and proper laboratory practice it is always should be possible to produce the standard curve of sufficient quality.

Specific design of standard curves may differ for different genes depending on the variability of their expression. For relatively stabile genes (*e.g*. Actin beta or GAPD) we usually were able to obtain good standard curves using 5–6 two-fold dilutions. To cover the dynamic range for genes with less stable expression (*e.g*. Mammaglobin 1 in breast cancers) more dilutions (up to 8) and/or higher factor at each dilution (3–5 fold) were needed. We usually run standards in triplicates (as well as the target specimens).

Even though the standard curves could be quite reproducible [12] we consider the presence of standard curves on each plate to be a good laboratory practice. Additionally, there is no great economy in sharing standard curves between PCR plates, when the plates are filled up with samples. For example, 6-point standard curve in triplicates takes just 18 cells: this is less than 20% of 96-plate and less than 5% of 386-plate. Therefore sharing of standard curves reduces costs and labour only in pilot experiments with small number of samples. However, even in pilot experiments the repeatability of shared standard curves should be validated on a regular basis.

#### Summarizing data from several reference genes

Several reference genes are required for accurate relative quantification [1,20]. Different ways may be used to derive a single normalizing factor out of several genes. To explore this in the attached version of VBA script we made available two options: arithmetic and geometric mean.

Arithmetic mean is the most "intuitive" way. However, it has a major disadvantage: it depends on arbitrary choice of the absolute values for reference genes. For example, the normalizing factor will differ, if a reference gene is described either as a fraction of 1 (absolute values from 0 to 1) or in percents (values 0% to 100%). Importantly, this can change the *relative *values of the normalizing factor in different samples. In contrast, if geometric mean is used, the arbitrary choice of units for any reference gene will not affect the relative values of normalizing factor in different samples. Neither arithmetic nor geometric mean accounts for differences in uncertainties of different reference genes. In practice this implies similar variances in all reference genes. This assumption seems reasonable in most of the cases. However, if this assumption does not hold the weights reciprocal to variances could be introduced.

Obviously, the different ways of summarizing data from reference genes will produce different results. At the same time, at truly stable expression of reference genes the general tendencies in results should be similar. Currently we calculate the single normalizing factor by geometric mean, because it better fits to the relative nature of measurements as well as to the logarithmic scale of gene expression changes [20,21].

Unfortunately common practice tends to ignore the uncertainty of normalizing factor. Our procedure estimates this uncertainty using the law of error propagation (see formulae in Additional file 1).

### Methods based on PCR efficiency and individual shapes of fluorescent plots

Standard curve approach was chosen for our procedure because currently PCR efficiency assessment may complicate data processing. The main complication is that actual efficiency of replication is not constant through the PCR run being high at exponential phase and gradually declining toward the plateau phase. However, most current methods of PCR efficiency assessment report "overall" efficiency as a single value. Additionally, PCR efficiency may be calculated in different ways that can "overestimate" or "underestimate" the "true" PCR efficiency [12]. In contrast, the standard curve method is based on a simple approximation of data obtained in standard dilutions to unknown samples.

At present the most popular method of PCR efficiency assessment is based on the slope of standard curve. This method does not account for PCR efficiencies in individual target samples. In contrast, recent publications on PCR efficiency assessment were concentrated on the analysis of individual shapes of fluorescence plots [8-10]. Potentially this may lead to better mathematical understanding of PCR dynamic and to new practical solutions in PCR quantification [13].

### Limitations of our data processing

This section summarizes conditions that must be adhered to in order to obtain valid results with our data processing:

• all PCRs must achieve doubtless plateau and no non-specific PCR products should be observed to use amplitude normalization;

• standard curves with coefficient of determination above 99% are required to ignore uncertainty of regression and to validate the use of amplitude normalization;

• low dispersion in PCR replicates (crossing points' CV < 1% or SD < 0.2) is required to use the conventional statistical methods.

These limitations are linked: amplitude normalization provides the low dispersion in replicas needed for statistical treatment.

## Conclusion

In this article we described a procedure for relative real time PCR data processing. The procedure is based on the standard curve approach, does not require PCR efficiency assessment, can be performed in fully automatic mode and provides statistical assessment of intra-assay PCR variation. The procedure has been carefully analyzed and tested. The standard curve approach was found a reliable and simple alternative to the PCR-efficiency based calculations in relative real time PCR.

## Methods

### Tissue samples, RNA extraction, reverse transcription

Breast cancer biopsies were taken from 21 patients before and after treatment with an aromatase inhibitor. Samples were obtained in the Edinburgh Breast Unit (Western General Hospital, Edinburgh) with patients' informed consent and ethical committee approval. Biopsies were snap frozen and stored in liquid nitrogen until RNA extraction. Before RNA extraction the frozen tissue was defrosted and stabilized in RNA-later-ICE reagent (Ambion). Total RNA was extracted with RNeasy-mini columns (Qiagen). Amount and purity of RNA were evaluated by spectrophotometer. RNA integrity was confirmed by agarose gel electrophoresis.

cDNA was synthesised with SuperScript III reverse transcriptase (Invitrogen) in accordance with the manufacturer's recommendations. Briefly:

1) oligo(dT)_20 _primers and dNTPs were added to total RNA,

2) the mix was heated to 65°C for 5 min and then chilled on ice,

3) first-Strand buffer, DDT, RNase inhibitor (RNaseOUT, Invitrogen) and Reverse transcriptase were added to specimens,

4) reverse transcription was carried out for 60 minutes at 50°C.

### PCR

**Figure 11:**
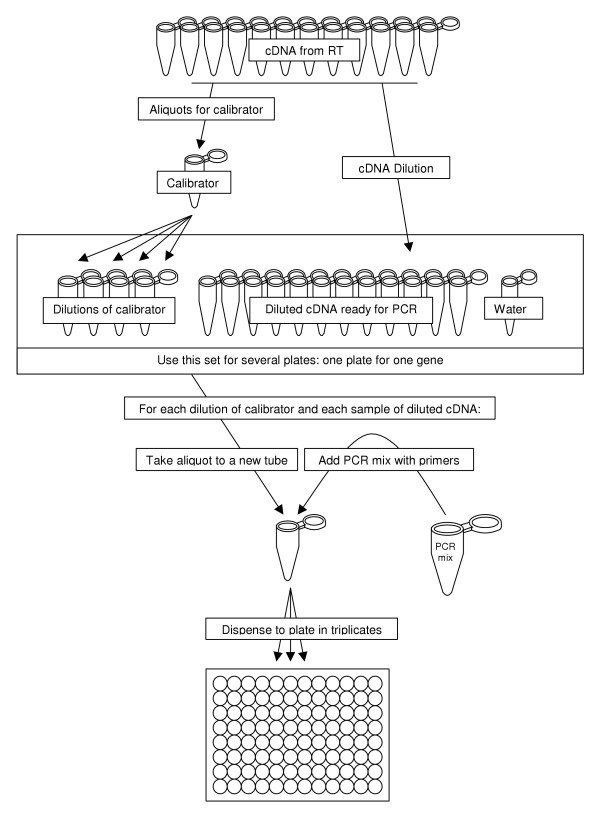
PCR set up.

Calibrator preparation, cDNA dilution and PCR plate set up were performed as illustrated in Figure 11. Briefly:

1. Aliquots of cDNA samples running on the same plate were pooled and the pool was used as calibrator.

2. cDNAs were diluted with water prior PCR.

3. The set of samples consisting of the diluted cDNAs and the dilutions of the calibrator were used for several PCR plates: one plate for each gene.

4. For each sample the whole PCR mix including primers and cDNA was prepared before dispensing into the plate.

5. Samples were loaded to 96× PCR plates by 15 μl per cell in triplicates or quadruplicates.

Primer's sequences are given in Table 1. Primers were designed basing on the sequences published in GenBank and using Primer-3 software [22]. To avoid genomic DNA amplification the primers were either located in different exons or across exon-exon boundaries. Primers were synthesized in Sigma Genosys or in Cancer Research UK. PCR was performed using QuantiTect SYBR Green PCR kit (Qiagen), Opticon-2 PCR machine (MJ Research), white 96× PCR plates and plain PCR caps (MJ Research). The cycling parameters for all genes were the following: hot-start 95°C 15 min, 45 cycles of (denaturation 94°C 15 sec, annealing 56°C 30 sec, elongation 72°C 30 sec, plate read), final elongation 72°C 5 min, melting curve 65–95°C. Gradient PCRs confirmed 56°C as appropriate annealing temperature for all primers.

Several additional PCRs were run with different amount of primers (0.1 μM, 0.3 μM, 0.9 μM), different amount of enzyme (0.8U, 1.5U and 3.1U of HotStarTaq, Qiagen were added to 15 μl PCRs made with QuantiTect SYBR Green PCR mix, Qiagen) and different caps (domed and plain caps, MJ Research).

### PCR product electrophoresis

Electrophoreses were run immediately after PCRs. 10 μl of PCR products were mixed with 2 μl of loading buffer. 6 μl of the mix per well was loaded into 10% PAAG (TBE Ready Gel, Biorad). Electrophoresis was run at 100 V for ~1 hr using MiniProtean-II cell (Biorad).

Prior electrophoresis 1 μl of 1:100 Sybr-Green-1 (Molecular Probes) was added into molecular weight marker (PCR Low Ladder Set, Sigma) but not into the PCR samples. After electrophoresis the gels were stained for 10 min in fresh prepared 1:10000 SybrGreen-1 (Molecular Probes). Photos were taken before and after staining using the GelDocMega4 gel documentation system (Uvitec).

### 96× PCR replicas

To study distributions of crossing points in PCR replicas four PCR plates have been run with a 96× replica on each. The distributions were evaluated using histograms, skewness and kurtosis measures, and the Kolmogorov-Smirnov test for Normality (see Table 2 and Figure 4).

### Normal distribution transformation through the data processing

**Figure 12:**
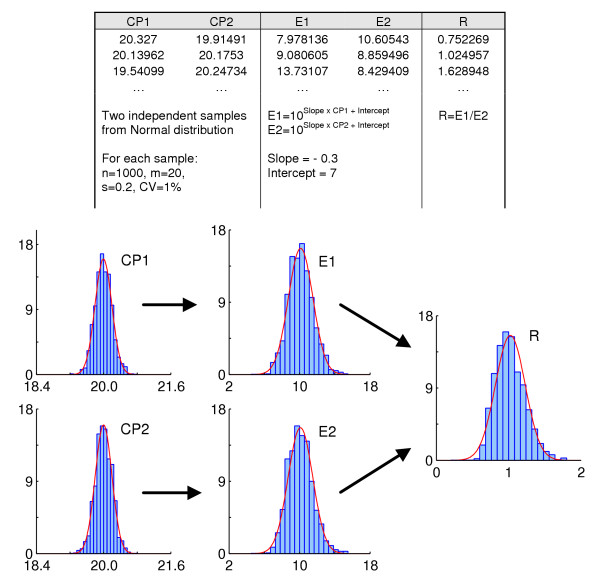
**Computer simulation of PCR data processing**. Computer simulation of PCR data processing at 1% CV in crossing points (see Methods for details).

The transformation of Normal distribution through data processing was studied by computer simulation (Figure 12).

Basing on the above empirical observations (Table 2, Figure 4) the crossing points were simulated by sampling from the Normal. Samples of 1,000 random normal numbers were obtained using standard Excel data analysis tool. A pair of such samples was used to simulate CPs for one target and one reference genes. Then the simulated CPs were processed in the same way as real PCR data. The distributions obtained at each step of data processing were evaluated for normality by histograms, skewness and kurtosis measures, and the Kolmogorov-Smirnov test.

Parameters used in calculations were close to actual parameters typically observed in our PCRs (MeanCP = 20, Slope = -0.3, Intercept = 7). The resulted true values for non-normalized and normalized results were 10 and 1 correspondingly.

To study error propagation at different initial dispersions we performed simulations using the Normal distributions with different variances (CV 0.5%, 1%, 1.5%, 2%, 3%, 4% and 5%; the means were always 20). Detailed illustration for CV 1% is presented in Figure 12. The summary of simulation results is presented in Figure 5 and Table 3.

### Excel VBA macros

The calculations where performed using MS Excel VBA script included to the electronic version of publication (see Additional file 2).

## List of abbreviations

*GAPD *– glyceraldehyde-3-phosphate dehydrogenase

CP (CPs) – crossing point (crossing points)

SD – standard deviation

CV – coefficient of variation

r^2 ^– coefficient of determination in linear regression

## Authors' contributions

AL carried out the main body of the project including PCR, statistics and programming.

WM conceived of the study and participated in its design and co-ordination.

AK verified statistical methods and mathematical calculations.

All co-authors contributed to the manuscript preparation.

## Supplementary Material

Additional File 1 Pdf file with formulae. Click here for file

Additional File 2 ZIP file containing VBA macros (PCR1.xls), test data for the above macros (Target1.csv, Target2.csv, Target3.csv, Target4.csv, Target5.csv, Reference1.csv, Reference2.csv) and instruction to the above macros (Instructions.pdf). Unzip file into a separate folder on your PC and follow the instructions. Click here for file

## Figures and Tables

**Table 1:** Primers' sequences

**Short name**	**Full name**	**GenBank number**	**Primers**
SCGB2A2	Mammaglobin 1 (Secretoglobin, family 2A, member 2)	NM_002411	TCC AAG ACA ATC AAT CCA CAA G AAA ATA AAT CAC AAA GAC TGC TG
SCGB2A1	Mammaglobin 2 (Secretoglobin, family 2A, member 1)	NM_002407	AAG ACC ATC AAT TCC GAC ATA CAC CAA ATG CTG TCG TAC ACT
CCNB1	Cyclin B1	NM_031966	CAT GGT GCA CTT TCC TCC TT CAG GTG CTG CAT AAC TGG AA
CKS2	CDC28 protein kinase regulatory subunit 2	NM_001827	TTC ATG AGC CAG AAC CAC AT CTC GTG CAC AGG TAT GGA TG
PTN	Pleiotrophin (heparin binding growth factor 8, neurite growth-promoting factor 1)	NM_002825	GTG CAA GCA AAC CAT GAA GA GCT CGC TTC AGA CTT CCA GT
LPIN2	Lipin 2	NM_014646	TTG TTG CTG CAG ATT GAT CC CCA AAT GGC AAT GGA TTT TC
ACTB	Actin, beta	NM_001101	GGA GCA ATG ATC TTG ATC TT CCT TCC TGG GCA TGG AGT CCT
GAPD	glyceraldehyde-3-phosphate dehydrogenase	NM_002046	TGC ACC ACC AAC TGC TTA GC GGC ATG GAC TGT GGT CAT GAG

Primers for *GAPD *were taken from Vandesompele *et al *[20]

**Table 2:** Crossing points' distributions observed in PCR replicas

**Plate**	**Number of replicates**	**Mean CP**	**SD**	**CV**	**Skewness**	**Kurtosis**	**Kolmogorov-Smirnov test**
1	96	21.48	0.06	0.3%	0.1	-0.1	Normal
2	94	18.09	0.07	0.4%	1.5	5.7	Sharper than normal
3	96	20.09	0.04	0.2%	0.1	-0.3	Normal
4	96	18.13	0.10	0.5%	0.5	1.0	Normal

**Table 3:** Magnitude of propagated error at different steps of data processing

**SD in crossing points**	**CV in crossing points**	**CV in non-normalized values**	**CV in normalized results**
0.1	0.5%	7%	10%
0.2	1.0%	14%	20%
0.3	1.5%	22%	31%
0.4	2.0%	28%	40%
0.6	3.0%	45%	66%

In all instances mean values are 20 in crossing points, 10 in non-normalized values and 1 in final results. See Figures 5 and 13 for more details.

## References

[B1] Bustin SA (2002). Quantification of mRNA using real-time reverse transcription PCR (RT-PCR): trends and problems. J Mol Endocrinol.

[B2] Muller PY, Janovjak H, Miserez AR, Dobbie Z (2002). Processing of gene expression data generated by quantitative real-time RT-PCR. Biotechniques.

[B3] Pfaffl MW (2001). A new mathematical model for relative quantification in real-time RT-PCR. Nucleic Acids Res.

[B4] Pfaffl MW, Horgan GW, Dempfle L (2002). Relative expression software tool (REST) for group-wise comparison and statistical analysis of relative expression results in real-time PCR. Nucleic Acids Res.

[B5] Livak KJ, Schmittgen TD (2001). Analysis of relative gene expression data using real-time quantitative PCR and the 2(-Delta Delta C(T)) Method. Methods.

[B6] Roshe Applied Science (2003). Overview of LightCycler Quantification Methods. Technical Note No LC 10.

[B7] Applied Biosystems (2004). Guide to Performing Relative Quantitation of Gene Expression Using Real-Time Quantitative PCR.

[B8] Tichopad A, Dilger M, Schwarz G, Pfaffl MW (2003). Standardized determination of real-time PCR efficiency from a single reaction set-up. Nucleic Acids Res.

[B9] Liu W, Saint DA (2002). A new quantitative method of real time reverse transcription polymerase chain reaction assay based on simulation of polymerase chain reaction kinetics. Anal Biochem.

[B10] Bar T, Stahlberg A, Muszta A, Kubista M (2003). Kinetic Outlier Detection (KOD) in real-time PCR. Nucleic Acids Res.

[B11] Rutledge RG, Cote C (2003). Mathematics of quantitative kinetic PCR and the application of standard curves. Nucleic Acids Res.

[B12] Pfaffl MW, Bustin SA (2004). Quantification strategies in real time PCR. A-Z of quantitative PCR.

[B13] Rutledge RG (2004). Sigmoidal curve-fitting redefines quantitative real-time PCR with the prospective of developing automated high-throughput applications. Nucleic Acids Res.

[B14] Wittwer CT, Herrmann MG, Moss AA, Rasmussen RP (1997). Continuous fluorescence monitoring of rapid cycle DNA amplification. Biotechniques.

[B15] Kainz P (2000). The PCR plateau phase - towards an understanding of its limitations. Biochim Biophys Acta.

[B16] Zipper H, Lämmle K, Buta C, Brunner H, Bernhagen J, Vitzthum F (2002). Investigations on the binding of SYBR Green I to double-stranded (ds)DNA: In Proceedings of the joint annual fall meeting , German Society for Biochemistry and Molecular Biology (GBM) & German Society for Expermental and Clinical Pharmacology and Toxicology (DGPT) September 7-10 2002; Halle (Saale), Germany..

[B17] Vitzthum F, Geiger G, Bisswanger H, Brunner H, Bernhagen J (1999). A quantitative fluorescence-based microplate assay for the determination of double-stranded DNA using SYBR Green I and a standard ultraviolet transilluminator gel imaging system. Anal Biochem.

[B18] Freshwater S, van der Valk A, O'Shaughnessy M, Ng S, Baker S, Pfaffl MW (2004). The effect of consumable type on the sensitivity and reproducibility of qPCR: In Proceedings of the 1^st^ International qPCR Symposium and Application Workshop 3rt - 6th March 2004; Freising-Weihenstephan, Germany..

[B19] Taylor BN, Kuyatt CE (1994). Guidelines for evaluating and expressing the uncertainty of NIST measurement results. NIST technical note ; 1297.

[B20] Vandesompele J, De Preter K, Pattyn F, Poppe B, Van Roy N, De Paepe A, Speleman F (2002). Accurate normalization of real-time quantitative RT-PCR data by geometric averaging of multiple internal control genes. Genome Biol.

[B21] Szabo A, Perou CM, Karaca M, Perreard L, Quackenbush JF, Bernard PS (2004). Statistical modeling for selecting housekeeper genes. Genome Biol.

[B22] Rozen S, Skaletsky HJ, Krawetz S and Misener S (2000). Primer3 on the WWW for general users and for biologist programmers. Bioinformatics Methods and Protocols: Methods in Molecular Biology.

[B23] Larionov AA, Hulme MJ, Miller WR, Pfaffl MW (2004). Amplitude normalization in real time PCR data processing: 3rt - 6th March 2004; Freising-Weihenstephan, Germany..

[B24] Larionov AA, Miller WR, Pfaffl MW (2004). Data processing in real time PCR: In Proceedings of the 1^st^ International qPCR Symposium and Application workshop Freising-Weihenstephan, Germany..

