# Supplementary material for: A standard curve based method for relative real time PCR data processing
Source: BMC Bioinformatics. 2005 Mar 21;6:62. doi: 10.1186/1471-2105-6-62 (PMC1274258; doi:10.1186/1471-2105-6-62)
Supplement: Additional File 1 — Pdf file with formulae. [file 1471-2105-6-62-S1.pdf]

Formulae

File content

Smoothing 2
Baseline subtraction 2
Amplitude normalization 2
Crossing points 2
Standard curve 3
Mean CPs and means' variances in replicates 3
Calculation of non-normalized values 3
Summarizing of reference genes 4
  Arithmetic mean 4
  Geometric mean 4
Calculation of final results 4
Calculation of confidence intervals 5
  Example of CI calculation 5
Appendix : Law of error propagation 6
  General form without covariance term 6
  Error propagation to non-normalized values 6
  Error propagation during summarizing of reference genes 6
    Arithmetic mean 6
    Geometric mean 7
  Error propagation through ratio 8

Smoothing

3 points moving average:

$$X_i = \frac{x_{i-1} + x_i + x_{i+1}}{3}$$

Where

X_i - smoothed value at i^{th} cycle and

x_i - value at i^{th} cycle before smoothing.

Note: For the 1st and last data point 2 points averages are used

Baseline subtraction

$$X_i = x_i - x_{\min}$$

Where

X_i - value at i^{th} cycle after baseline subtraction,

x_i - value at i^{th} cycle before baseline subtraction and

$x_{\min}$ - minimal value in the cell through the whole PCR run.

Amplitude normalization

$$X_i = \frac{x_i}{x_{\max}}$$

Where

X_i - value at i^{th} cycle after amplitude normalization,

x_i - value at i^{th} cycle before amplitude normalization and

$x_{\max}$ - maximal value in the cell through the whole PCR run.

Crossing points

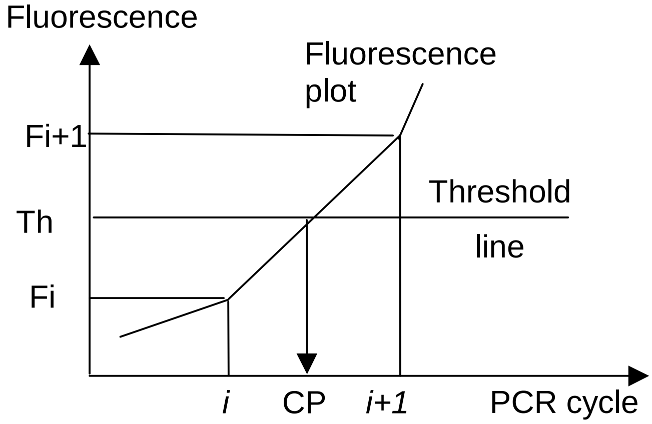

Direct calculation:

$$CP = i + \frac{Th - F_i}{F_{i+1} - F_i}$$

Were

CP - Crossing point value

Th - Threshold value

F_i - fluorescence value at i^{th} cycle and

i^{th} cycle is the last cycle in the run where $F_i < Th < F_{i+1}$

Standard curve

Least squares linear regression

$$\hat{y}_i = ax_i + b$$

$$a = \frac{\sum (x_i - \bar{x})(y_i - \bar{y})}{\sum (x_i - \bar{x})^2}$$

$$b = \bar{y} - a\bar{x}$$

$$r^2 = \frac{\sum (\hat{y}_i - \bar{y})^2}{\sum (y_i - \bar{y})^2}$$

Where

a - slope, b - intercept,

r^2 - coefficient of determination (squared coefficient of correlation)

x_i and y_i - observed values, $\bar{x}$ and $\bar{y}$ - mean values, $\hat{y}_i$ - predicted values

Mean CPs and means' variances in replicates

Arithmetic mean

$$\overline{CP} = \frac{\sum_{i=1}^{n} CP_i}{n}$$

$$s^2_{\overline{CP}} = \frac{\sum (CP_i - \overline{CP})^2}{n(n-1)}$$

Were

$\overline{CP}$ and $S^2_{\overline{CP}}$ are mean CP and its variance,

CP_i - individual CPs in replicates,

n – number of replicates.

Calculation of non-normalized values

Non-normalized values were calculated from mean crossing points using linear function (standard curve) followed by exponent. Variances of non-normalized values were estimated by the law of error propagation (see Appendix at the end of this file).

Best fit values

$$\overline{X} = 10^{Slope \times \overline{CP} + Intercept}$$

Error propagation

$$S^2_{\overline{X}} = Slope^2 \times s^2_{\overline{CP}} \times (\ln 10 \times 10^{Slope \times \overline{CP} + Intercept})^2$$

Where

$\overline{X}$ and $S^2_{\overline{X}}$ are the best estimate of non-normalized value and its variance,

$\overline{CP}$ and $s^2_{\overline{CP}}$ - mean CP in replicates and its variance,

Slope and *Intercept* are parameters of the standard curve.

Summarizing of reference genes

For arithmetic and geometric means the propagated variances were estimated by the law of error propagation (see Appendix at the end of this file).

Arithmetic mean

Best fit values

$$\bar{x} = \frac{\sum x_i}{n}$$

Error propagation

$$s_{\bar{x}}^2 = \frac{\sum s_{xi}^2}{n^2}$$

Where

$\bar{x}$ and $s_{\bar{x}}^2$ are arithmetic mean of reference genes and its variance

x_i and s_{xi}^2 are the best-fit value and its variance for each reference gene

Geometric mean

Best fit values

$$\bar{x} = \sqrt[n]{x_i \times \ldots \times x_n}$$

Error propagation

$$s_{\bar{x}}^2 = \sum \left[\left(\frac{\sqrt[n]{x_i \times \ldots \times x_n}}{n \times x_i} \right)^2 \times s_{xi}^2 \right]$$

Where

$\bar{x}$ and $s_{\bar{x}}^2$ are geometric mean of reference genes and its variance

x_i and s_{xi}^2 are the best-fit value and its variance for each reference gene

Calculation of final results

Final results (relative expressions of target genes) were calculated dividing non-normalized values of target genes by the normalization factor derived from reference genes. Variance if the final results was estimated by the low of error propagation (see Appendix at the end of this file)

Best fit values

$$X = \frac{T}{R}$$

Error propagation

$$S_X^2 = \frac{1}{R^2} \times \left(S_T^2 + \frac{T^2 \times S_R^2}{R^2} \right)$$

Were

X – Final normalized result (e***X***pression)
T - Non-normalized value for a target gene (***T***arget)
R - Single normalizing factor calculated from several reference genes (***R***eference)
S_X^2 - variance of *X*
S_T^2 - variance of *T*
S_R^2 - variance of *R*

Calculation of confidence intervals

95% confidence intervals were calculated from estimated variances.

$$CI = t \times \sqrt{s^2}$$

Were

CI - confidence interval

t - coefficient for 95% confidence intervals from appropriate t-distribution (corresponding to the number of degrees of freedom)

s^2 - variance

The number of degree of freedom (DF) was calculated basing on the number of replicas in all genes used for confidence interval (see example below). Genes were considered fixed factors.

Example of CI calculation

This illustrates calculation of the t-coefficients for the case when relative expression of a target gene was calculated using two reference genes and all PCR were done in quadruplicates.

**Gene**	**Number of replicas**	**Degrees of freedom**
Target	4	3 = 4 - 1
Reference1	4	3 = 4 - 1
Reference2	4	3 = 4 - 1

**Confidence interval**	**Degrees of freedom**	**Coefficient t**
Non-normalized values	3<br>= DF for each individual gene	3.182449
Normalizing factor	6<br>= DF(Reference1)<br>+ DF(Reference2)	2.446914
Final result	9<br>= DF(Reference1)<br>+ DF(Reference2)<br>+ DF(Traget)	2.262159

Appendix : Law of error propagation

General form without covariance term

$$S_Y^2 = \sum\left[\left(\frac{\partial F(X_1,...,X_n)}{\partial X_i}\right)^2 \times S_{Xi}^2\right]$$

Where

$Y = F(X_1,...,X_n)$

S_Y^2 - variance of Y

S_{Xi}^2 - variance of Xi

Error propagation to non-normalized values

Non-normalized values were calculated by linear function followed by decimal exponent:

$$Y = 10^{aX+b}$$

First derivative:

$$\frac{\partial Y}{\partial X} = a \times \ln 10 \times 10^{aX+b}$$

Propagated variance:

$$S_Y^2 = \left(a \times \ln 10 \times 10^{aX+b}\right)^2 \times S_X^2$$

Error propagation during summarizing of reference genes

Arithmetic mean

$$Y = \frac{X_1 + ... + X_n}{n}$$

Partial derivatives:

$$\frac{\partial Y}{\partial X_i} = \frac{\partial\left[\frac{X_1 + ... + X_n}{n}\right]}{\partial X_i} = \frac{1}{n}$$

Propagated variance:

$$S_Y^2 = \frac{\sum S_{Xi}^2}{n^2}$$

Geometric mean

$$Y = \sqrt[n]{X_1 \times ... \times X_n}$$

Partial derivatives:

$$\frac{\partial Y}{\partial X_i} = \frac{\partial \left[X_i^{\frac{1}{n}} \times \sqrt[n]{X_1 \times ... \times X_{i-1} \times X_{i+1} \times ... \times X_n} \right]}{\partial X_i}$$

$$= \frac{1}{n} \times X_i^{\frac{1}{n}-1} \times \sqrt[n]{X_1 \times ... \times X_{i-1} \times X_{i+1} \times ... \times X_n}$$

$$= \frac{1}{n} \times X_i^{\frac{1}{n}-1} \times \frac{\sqrt[n]{X_1 \times ... \times X_{i-1} \times X_i \times X_{i+1} \times ... \times X_n}}{\sqrt[n]{X_i}}$$

$$= \frac{1}{n} \times X_i^{\frac{1}{n}-1} \times \frac{\sqrt[n]{X_1 \times ... \times X_n}}{\sqrt[n]{X_i}}$$

$$= \frac{1}{n} \times X_i^{\frac{1}{n}-1} \times X_i^{-\frac{1}{n}} \times \sqrt[n]{X_1 \times ... \times X_n}$$

$$= \frac{1}{n} \times X_i^{-1} \times \sqrt[n]{X_1 \times ... \times X_n}$$

$$= \frac{\sqrt[n]{X_1 \times ... \times X_n}}{n \times X_i}$$

Propagated variance:

$$S_Y^2 = \sum \left[\left(\frac{\sqrt[n]{X_1 \times ... \times X_n}}{n \times X_i} \right)^2 \times S_{Xi}^2 \right]$$

Error propagation through ratio

$$Y = \frac{X_1}{X_2}$$

Partial derivatives:

$$\frac{\partial Y}{\partial X_1} = \frac{1}{X_2}$$

$$\frac{\partial Y}{\partial X_2} = -\frac{X_1}{X_2^2}$$

Propagated variance:

$$S_Y^2 = \left(\frac{1}{X_2}\right)^2 \times S_{X_1}^2 + \left(\frac{X_1}{X_2^2}\right)^2 \times S_{X_2}^2$$

$$= \frac{S_{X_1}^2}{X_2^2} + \frac{X_1^2 \times S_{X_2}^2}{X_2^4}$$

$$= \frac{1}{X_2^2} \times \left(S_{X_1}^2 + \frac{X_1^2 \times S_{X_2}^2}{X_2^2} \right)$$
