# Supplementary material for: A standard curve based method for relative real time PCR data processing
Source: BMC Bioinformatics. 2005 Mar 21;6:62. doi: 10.1186/1471-2105-6-62 (PMC1274258; doi:10.1186/1471-2105-6-62)
Supplement: Additional File 2 — ZIP file containing VBA macros (PCR1.xls), test data for the above macros (Target1.csv, Target2.csv, Target3.csv, Target4.csv, Target5.csv, Reference1.csv, Reference2.csv) and instruction to the above macros (Instructions.pdf). Unzip file into a separate folder on your PC and follow the instructions. [file 1471-2105-6-62-S2.zip › Instructions.pdf]

Instruction for the script

File content

Introduction 1
Instructions 1
If you use provided examples of source data 1
If you use your own data 2
Input files: PCR1 format 2
Output files 3
Notes 4

Introduction

This is instruction for the following files::

Files	Brief description
PCR1.xls	Excel file with macros in it.
TARGET1.csv<br>TARGET2.csv<br>TARGET3.csv<br>TARGET4.csv<br>TARGET5.csv<br>REFERENCE1.csv<br>REFERENCE 2.csv	Example of files with source data that can be used with the above script. These are CSV files in PCR1 format with actual data obtained in real time PCR for five target and two reference genes.

All above files are provided solely to illustrate the concepts presented in the article (A.Larionov *et al* 2005, BMC Bioinformatics).

The script was written for personal use, it is published "as is", no warranties or technical support is assumed. However, questions and suggestions may be addressed to the corresponding author. In the author's hands the above script and source data worked in Excel 2002 under Windows XP as well as in Excel 97 under Windows NT. The script was not optimised for speed, so it may take some minutes to execute it in a slow machine.

If you use the script with your own data keep the input sensible: *e.g.* no negative values, no zero concentrations in standards, no identical labels in different samples *etc.*. Most probable, the wrong input will lead to an error termination. However, beware of strange and unexpected results – double-check your input in such case.

Instructions

If you use provided examples of source data

1. Copy the file with script into an empty folder.
2. Copy data files to the same folder.
3. Ensure enough space in the hard drive to allocate all output files (~ 1 Mb per gene).
4. Launch the script file (allow macros).
5. Select reference and studied genes, select analysis options.

6. Click "Calculate" button and follow the instructions appearing on dialogues (if any).

If you use your own data

1. Using your cycler's software export raw data to Excel-compatible file format.
   - Make sure that you export ***raw*** readings: *i.e.* no baseline subtraction, smoothing *etc.* has been applied to the fluorescence readings.
   - Do not export obvious outliners (*e.g.* samples were fluorescence plot has non-sigmoidal shape). You will not be able to exclude them later.
   - Do not export samples with no PCR products in standards and reference genes.
2. Using MS Excel manually cast data into PCR1 file format (see below).
3. Follow steps described above for the provided examples of source data.

Input files: PCR1 format

The script accepts data in form of CSV file with the following content:

File Format label →

PCR1	A1	A2	A3	A4	A5	…
	Standard	Standard	Blank	Sample	Sample	
	100	50	Water	Patient1	Patient2	
1	0.157	0.1324	0.1325	0.1662	0.1316	
2	0.1542	0.1324	0.133	0.1687	0.1321	
3	0.1569	0.132	0.1336	0.167	0.1339	
4	0.156	0.1347	0.1321	0.1627	0.1318	
…						…

← Comments (row 1)
← Sample types (row 2)
← Sample labels (row 3)
Cycle numbers (first column, rows 4 onward)
Fluorescence readings (table body)

1. File format label "PCR1" must be placed in the top left cell.
2. The first row of the table is used for comments (except the left cell used for "PCR1" label). Phrase "No fluorescence rise" is not allowed in comments. Except this phrase the comments will not be analysed. In the above example the comments are used to store cell co-ordinate in the plate.
3. The second row is used for sample type (except the left cell that is not used). Only three types of samples are allowed: "Standard", "Sample" or "Blank". If script detects unknown sample type it interprets it as an error and terminates calculations.
4. The third row is used for sample label (except the left cell that is not used). No empty labels allowed (even in blanks)! Labels of standards are their values and so must be numerical. In contrast, the labels of samples will be sorted as text. So it is better to start them with a letter and keep leading zeros in numerical indexes (if any). Avoid labels that can be interpreted and sorted as dates.
5. The first column (except the top three cells) is used for cycle numbers. It is assumed that just one reading is taken at each cycle. Cycle numbers must be integer numbers.
6. The table body is used for fluorescence readings that must be positive numbers.

Output files

The following output files will be generated by script:

Files	Content	Location	Name
Summary report for all genes	Summary of all non-normalized values, normalizing factor and relative expressions	In the script's folder	PCR Results *date time*.xls
Individual files for each gene	Detailed calculations for non-normalized values for each gene separately	In the folders were the original CSV files are	* *date time*.xls<br>were<br>* is name of the source file

The detailed content of output files:

<table>
<tr><th>Files</th><th>Worksheets and charts</th><th>Content</th></tr>
<tr><td rowspan="9">PCR Results
date time.xls

Summary report for all genes</td><td>Notes</td><td>List of input, output files and analysis options</td></tr>
<tr><td>Target Genes</td><td>Non-normalized values for target genes</td></tr>
<tr><td>Reference Genes</td><td>Non-normalized values for reference genes</td></tr>
<tr><td>Reference Genes Chart</td><td rowspan="2">Charts for reference genes:
blue – reference genes
red – normalizing factor</td></tr>
<tr><td>Reference Genes Log Chart</td></tr>
<tr><td>Relative Gene Expressions</td><td>Relative expressions of target genes</td></tr>
<tr><td>Target1 date time</td><td rowspan="3">Charts for relative expressions of target genes</td></tr>
<tr><td>Log Target1 date time</td></tr>
<tr><td>etc</td></tr>
<tr><td rowspan="17">* date time.xls

were
* is name of the source file

Detailed calculations for non-normalized values for each gene separately</td><td>PCR1 Format</td><td>Source data</td></tr>
<tr><td>Sort</td><td>Source data sorted by type and label</td></tr>
<tr><td>Sort Chart</td><td>Chart of sorted data (standards only)</td></tr>
<tr><td>Smooth</td><td>Data after smoothing</td></tr>
<tr><td>Smooth Chart</td><td>Chart of smoothed data (standards only)</td></tr>
<tr><td>Baseline</td><td>Data after baseline subtraction</td></tr>
<tr><td>Baseline Chart</td><td>Chart of baseline subtracted data (standards only)</td></tr>
<tr><td>Amplitude</td><td>Data after amplitude normalization (optional)</td></tr>
<tr><td>Amplitude Chart</td><td>Chart of amplitude normalized data
(standards only, optional)</td></tr>
<tr><td>Threshold</td><td>Data for threshold selection</td></tr>
<tr><td>Standard Curve</td><td>Chart for standard curve
(with confidence and prediction bars)</td></tr>
<tr><td>Crossing Points</td><td>Data for crossing points</td></tr>
<tr><td>Results</td><td>Non-normalized values for the gene</td></tr>
<tr><td>Results Chart</td><td rowspan="2">Charts for non-normalized values</td></tr>
<tr><td>Results Log Chart</td></tr>
</table>

Notes

1. Each output file unlikely exceeds 1 MB. Enough space in the hard drive must be available to allocate all output files. The script does not check space on the hard drive. Insufficient disk space will cause a run time error.
2. Absolute fluorescence value in the last cycle must exceed the fluorescence in the first cycle by at least 10%. Otherwise the PCR products are considered undetectable.
3. Failure to detect PCR products in any standard will prevent further calculations
(in other words: expression of ALL genes must be detectable in ALL standards).
4. Failure to detect a reference gene expression may prevent relative quantification
(in other words: expression of ALL reference genes should be detectable in ALL samples).
5. For target genes the undetectable PCR products are allowed in samples but not in standards.
6. For relative quantification the labels of samples must be exactly the same in target and reference genes and no samples can be missed
(in other words: for relative quantification exactly the same set of samples must be run for all target and reference genes).
7. For single measurements no statistical assessment of intra-assay variation can be done. In most of cases script will work with such data leaving correspondent fields empty. However, it was intended to work with replicates (we tested it mostly with triplicates).
8. Search for optimal threshold is limited to
   - lower half of fluorescence plot if amplitude normalization is used,
   - lower third of fluorescence plot if amplitude normalization is not used.
9. The following rare issues were found in testing:
   - Excessive plateau scattering can cause error termination if amplitude normalization is not used ("No PCR product in one of the standards" error occur if more than 3 times difference in the plateau positions is observed after baseline subtraction).
   - Very high dispersion in replicas and very low best-fit value may result to a confidence limit below zero. This causes minor problems in log-scale charts generated in output files.
10. In case of any errors – delete all output files, check source data, try to find out and fix the problem. Then start from the beginning.
